# Microbially-produced folate forms support the growth of *Roseburia intestinalis* but not its competitive fitness in fecal batch fermentations

**DOI:** 10.1186/s12866-024-03528-6

**Published:** 2024-09-28

**Authors:** Palni Kundra, Annelies Geirnaert, Benoit Pugin, Serafina Plüss, Susanna Kariluoto, Christophe Lacroix, Anna Greppi

**Affiliations:** 1 Department of Health Science and Technology, ETH Zurich, Institute of Food, Nutrition and Health, Laboratory of Food Biotechnology, Schmelzbergstrasse 7, Zurich, 8092 Switzerland; 2 Department of Food and Nutrition, University of Helsinki, Agnes Sjöbergin Katu 2, 00014 Helsinki, Finland; 3 Present Address: Department of Health Science and Technology, ETH Zurich, Institute of Food, Nutrition and Health, Laboratory of Food Systems Biotechnology, Schmelzbergstrasse 7, Zurich, 8092 Switzerland

**Keywords:** Vitamin B9, THF, M-THF, F-THF, FFA, Folic acid, Prototrophs, Fecal fermentations

## Abstract

**Background:**

Folate (vitamin B9) occurs naturally mainly as tetrahydrofolate (THF), methyl-tetrahydrofolate (M-THF), and formyl-tetrahydrofolate (F-THF), and as dietary synthetic form (folic acid). While folate auxotrophy and prototrophy are known for several gut microbes, the specific folate forms produced by gut prototrophs and their impact on gut auxotrophs and microbiota remain unexplored.

**Methods:**

Here, we quantified by UHPLC-FL/UV folate produced by six predicted gut prototrophs (*Marvinbryantia formatexigens* DSM 14469*, Blautia hydrogenotrophica* 10507 ^T^*, Blautia producta* DSM 14466, *Bacteroides caccae* DSM 19024, *Bacteroides ovatus* DSM 1896*,* and *Bacteroides thetaiotaomicron* DSM 2079 ^T^) and investigated the impact of different folate forms and doses (50 and 200 µg/l) on the growth and metabolism of the gut auxotroph *Roseburia intestinalis* in pure cultures and during fecal anaerobic batch fermentations (48 h, 37 °C) of five healthy adults.

**Results:**

Our results confirmed the production of folate by all six gut strains, in the range from 15.3 ng/ml to 205.4 ng/ml. Different folate forms were detected, with THF ranging from 12.8 to 41.4 ng/ml and 5-MTHF ranging from 0.2 to 113.3 ng/ml, and being detected in all strains. Natural folate forms, in contrast to folic acid, promoted the growth and metabolism of the auxotroph *R. intestinalis* L1-82, with dose-dependent effects. During fecal batch fermentations, folate forms at both levels had no detectable effect on total bacteria concentration, on gut community composition and metabolic activity and on *Roseburia* spp. abundance, compared to the control without folate addition.

**Conclusions:**

Our study demonstrates for the first time in vitro the production of different natural folate forms by predicted gut prototrophs and the stimulation on the growth of the folate auxotrophic butyrate-producing *R. intestinalis* L1-82. Surprisingly, folate did not impact fecal fermentations. Our data suggest that the dietary folate forms at the tested levels may only have limited effects, if any, on the human gut microbiota in vivo.

**Supplementary Information:**

The online version contains supplementary material available at 10.1186/s12866-024-03528-6.

## Introduction

Folate (vitamin B9) is an umbrella term for water-soluble B-vitamins, referring to a diverse group of molecules with three chemical components in common: i) a pteridine ring, which can be reduced or oxidized; ii) a para-aminobenzoic acid (PABA) connected to the pteridine ring, and iii) a polyglutamate tail with a varying chain length [1]. Different natural forms of reduced folates, (e.g. polyglutamated 5-methyltetrahydrofolate (M-THF)), are found in foods like cereals, berries and green leafy vegetables [2, 3] while the synthetic oxidized folic acid is mainly used as dietary supplement or for food fortification. Compared to synthetic folic acid, M-THF offers significant advantages in terms of absorption rates, retaining its effectiveness even when gastrointestinal pH levels are altered. Additionally, its bioavailability remains unaffected by metabolic defects [4]. The impact of various forms of folate on gut bacteria and microbiota, both at the individual and community levels, is yet to be elucidated.

In bacteria, folate serves as a crucial cofactor in one-carbon unit metabolism, participating in essential cellular processes such as serine, thymidylate, purines, and methionine synthesis, as well as in the acetyl-CoA pathway, an important catabolic route in acetogens known as the Wood–Ljungdahl pathway [5, 6]. Since folate biosynthesis is metabolically costly, involving a complex pathway with 11 functional genes [7], it is hypothesized that within microbial communities like the human gut microbiome, folate is frequently exchanged between producers (prototrophs) and non-producers (auxotrophs) [8, 9]. Indeed, several studies have reported the potential of human gut microbial taxa to synthesize folate [7, 10, 11]. However, the in vitro demonstration of the production of diverse folate forms by prototrophic gut microbes is still lacking.

Within the human gut microbiota community, increased intake of folate was positively associated with a higher abundance of *Faecalibacterium*, *Akkermansia* and *Roseburia* genus, as observed in mucosal and colonic biopsy samples [12]. Notably, *Roseburia intestinalis* is a prevalent butyrate producer of the healthy adult human gut microbiota, ranking among the top 20 most abundant species with an average relative abundance of 2.3% [13]. *R*. *intestinalis* has been recently proposed as a potential biomarker of gut health and a candidate for next-generation probiotic in live biotherapeutic products due to its association with a healthy gut and its correlation with improved outcomes in conditions such as atherosclerosis, alcohol-related liver diseases, colitis, and colorectal cancer [14]. Genomic analysis indicates that *R. intestinalis* lacks the capability to synthesize folate [15], a finding confirmed in vitro [16]. Furthermore, its abundance was found to be decreased in diseases associated with lower dietary folate intake and biosynthesis, such as Parkinson’s disease and colitis [6, 17–19]. Recently, it was demonstrated that three folate gut prototrophs can support the growth of *R. intestinalis* when grown in co-cultures [16]; however, the specific forms of folate were neither identified nor quantified in this interaction. Further, it remains unclear whether *R. intestinalis* can utilize all different forms of folate produced by prototrophs, and what effect these forms have on its growth kinetics. Lastly, the role of microbially-produced folate forms in providing a competitive advantage for *R. intestinalis* within the human gut microbiota remains also to be explored.

In this study, we aimed to identify and quantify folate forms produced by six predicted gut prototrophs during batch fermentations and investigate the impact of different folate forms in inactivated prototroph extracts on the *R. intestinalis* growth during pure batch cultures. Last, we tested the potential effects of commercially available folate forms and folic acid during fecal anaerobic batch fermentations of five healthy adults.

## Material and methods

### Bacterial strains and cultivation conditions

A panel of six putative folate prototrophic strains, namely *Marvinbryantia formatexigens* DSM 14469*, Blautia hydrogenotrophica* 10507^ T^*, Blautia producta* DSM 14466, *Bacteroides caccae* DSM 19024, *Bacteroides ovatus* DSM 1896*,* and *Bacteroides thetaiotaomicron* DSM 2079^ T^, identified from in silico genomic data [7, 11] was used in this study. *R. intestinali*s L1-82 was selected as butyrate-producing folate auxotrophic strain [16]. All strains were acquired from the German Collection of Microorganisms and Cell Culture GmbH (DSMZ, Braunschweig, Germany). Strains were stored at − 80 °C in 25% (v/v) anaerobic glycerol stocks, and they were routinely cultivated in basal medium consisting of (in l^−1^; all chemicals purchased from Sigma-Aldrich Chemie GmbH, Buchs, Switzerland, unless otherwise stated): 20 g tryptone (Chemie Brunschwig AG, Basel, Switzerland), 4 g L-threonine, 0.5 mg resazurin (redox indicator), 4 g NaHCO_3_, 1 g L-cysteine HCl, 0.4 g KH_2_PO_4_, 0.53 g Na_2_HPO_4_, 0.3 g NH_4_Cl, 0.3 g NaCl, 0.1 g MgCl_2_‧6H_2_O, 0.11 g CaCl_2_, 1 ml of acid stock solution (contains per liter 50 mM HCl (VWR International AG, Dietikon, Switzerland), 1 mM H_3_BO_3_, 0.5 mM MnCl_2_, 7.5 mM FeCl_2_, 0.5 mM CoCl_2_, 0.1 mM NiCl_2_, and 0.5 mM ZnCl_2_), 1 ml of alkaline stock solution (contains per liter: 10 mM NaOH, 0.1 mM Na_2_SeO_3_, 0.1 mM Na_2_WO_4_, and 0.1 mM Na_2_MoO_4_) [20]. The medium was supplemented with vitamins (per liter): 20 µg biotin, 20 µg nicotinic acid, 10 µg p‐aminobenzoic acid, 20 µg thiamine, 20 µg pantothenate, 50 µg pyridoxamine, 10 µg cyanocobalamin, 20 µg folic acid, 10 µg riboflavin, 5 µg lipoic acid, 10 µg menadione and 0.75 µg phylloquinone [21]. The basal medium was supplemented either with formate (50 mM) for formate-utilizing strains (*M. formatexigens* and *B. hydrogenotrophica*), or with acetate (33 mM; Chemie Brunschwig AG) for all other strains. For all the strains, glucose (27 mM) was supplemented. The medium was prepared anaerobically using the Hungate technique as previously described [22].

Before performing each experiment, bacterial cultures were reactivated from glycerol stocks and sub-cultured at least twice before being used for the culture experiments inoculum (Hungate tubes, CO_2_ headspace, 2% (v/v) inoculum, incubated for 48 h at 37 °C). Moreover, samples were taken from the inoculum for Gram staining to confirm the purity of the cultures.

### Preparation of natural folate in inactivated bacterial extracts

Inactivated bacterial (IB) extracts (fermented medium containing heat lysed cell) were prepared from folate prototrophic species (*M. formatexigens,* MFP; *B. hydrogenotrophica,* BHP; *B. producta,* BPP; *B. caccae,* BCP; *B. ovatus,* BOP; and *B. thetaiotaomicron,* BTP). To avoid any folate carryover from the medium, all six folate prototrophic strains were sub-cultured twice in a chemically defined medium (CDM), deprived of folate and formulated for the cultivation of human gut anaerobes [16]. Pre-cultures were prepared anaerobically in Hungate tubes (2% (v/v) inoculum, incubated for 48 h at 37 °C) as described above, and further incubated into serum flasks for about 60 h in 50 ml CDM with folate precursor PABA since several folate prototrophic microbes are known to be auxotroph for PABA [11]. Total bacteria and SCFA were measured using 1 ml of fermented medium, as described below. The remaining 48 ml of fermented medium was used for the preparation of IB extracts. The cells were lysed by heating the fermented medium in 10 ml falcon tubes in a water bath at 100 °C for 10 min and vortexed twice during heating. The samples were then centrifuged at 12′000 × g for 10 min at 4 °C to remove cell debris. Sterile filtered (0.2 µm, VWR International AG) supernatants were transferred into nitrogen pre-flushed sterile Hungate tubes covered with aluminum foil for light protection and stored in -80 °C freezers until further analysis. All processing was carried out in dark conditions to prevent folate degradation from light.

### Growth of *R*. *intestinalis* L1-82 with IB extracts from folate prototrophs

To investigate whether *R. intestinalis* L1-82 can use folate in IB extracts of prototrophic strains, we conducted the growth test of *R. intestinalis* L1-82 strain in 96-deepwell plates (Ritter GmbH, Schwabenmünchen, Germany). Before performing each experiments, *R. intestinalis* L1-82 precultures were prepared as described above and the optical density (OD_600_) of pre cultures was then normalized to 1 by diluting with anaerobic phosphate buffer saline (PBS) composed of (in l^−1^; all from Sigma-Aldrich Chemie GmbH unless otherwise stated): 8.8 g K_2_HPO_4_; 6.8 g KH_2_PO_4_ (VWR International AG); 8 g sodium chloride; 1 g L-cysteine hydrochloric acid; 1 ml of 0.05% resazurin stock solution. Cells were washed three times with anaerobic PBS using MiniSpin centrifuged at 5000 g for 10 min. The resulting cell suspension (30 µl) was added to 0.6 ml of two-fold concentrated CDM with or without added folates and mixed with 0.6 ml of each IB extracts. The plate was sealed with a breathable seal (Breathe-Easier Sealing film, Diversified Biotech, Dedham, Massachusetts, USA) and incubated at 37 °C for 48 h in an anaerobic chamber (10% CO_2_, 5% H_2_, and 85% N_2_; Coy Laboratories, Grass Lake, Michigan, USA) as previously described [23]. After 48 h, the pH was measured directly in the deep-well plates using a pH meter (Metrohm Switzerland Ltd, Zofingen, Switzerland). The OD_600_ was measured at 600 nm with 150 µl samples using a plate reader (BioTek, PowerWave XS; BioTek Instruments, Inc., Vermont, USA). Afterwards, the 96-deepwell plates were centrifuged for 10 min (5000 × g, 4 °C) and the supernatants (1 ml) were transferred to a new 96-deepwell plate, sealed with clear seal (VWR International AG), and stored at –20 °C, until further analysis. Three biological replicates, each in technical duplicate, were performed.

### Growth of *R*. *intestinalis* L1-82 with specific chemical folate forms

*R. intestinalis* L1-82 was also cultivated in CDM in 96-well microtiter plates (300 µl, Milian SA) containing 180 µl of CDM medium supplemented with 20 µl of stock solutions containing different forms of commercial folates: tetrahydrofolate, THF; 5-methyltetrahydrofolate, M-THF; 10-formylfolic acid, FFA; 5-formyltetrahydrofolate, F-THF (Schircks Laboratories, Rapperswil-Jona, Switzerland), or folic acid (VWR International AG) with final concentration of 50 µg/l. Outer wells of the plates were filled with 200 µl of filtered water to prevent evaporation during incubation. Washed precultures of *R. intestinalis* L1-82 (30 µl) were inoculated at 2.5% (v/v) and the plate was incubated at 37 °C in the microplate reader (Tecan Trading, Mannerdorf, Switzerland) directly placed in an anaerobic chamber and OD was measured every 20 min during 70 h. The maximum specific growth rates (µ_max_; h^−1^) were estimated using linear regression analysis on the linear period of the exponential growth phase determined by plotting log_10_ OD versus time (R^2^ > 0.99). Then µ_max_ (h^−1^) was estimated from the slope of the regression line multiplied by Ln 10.

### Fecal sample collection and preparation

Fecal samples were collected from five healthy adult donors, two females (D1, D3) and three males (D2, D4 and D5), with median age of 28 years and no antibiotic intake in the previous 3 months. Feces were sampled directly after defecation using a sterile container and placed in an anaerobic atmosphere generator (Anaerogen; Thermo Fisher Diagnostics AG, Pratteln, Switzerland) and conveyed into an anaerobic chamber in less than 1 h. A 10% (w/v) fecal suspension was prepared in sterile anaerobic PBS (pH 6.5). The suspension was homogenized by pipetting up-down, let stand for 5 min to allow particles to settle to the bottom. The fecal slurry was serially tenfold diluted to 10^-4^ dilution before inoculation.

### Fecal batch fermentations

Fecal batch fermentations were performed in 96-deepwell plates in basal YCFA (bYCFA) medium adapted for batch fermentations (pH 6.8) as previously described [23]. Composition of the bYCFA medium is as follows (l^−1^): 1.0 g amicase, 1.25 g yeast extracts, 0.5 g meat extracts, 3.0 g soluble starch (used as a carbon source), 0.01 g hemin, 0.001 g resazurin sodium salt, 4.0 g NaHCO_3_, 0.9 g NaCl, 0.525 g KH_2_PO_4_, 0.9 g (NH_4_)_2_SO_4_, 0.09 g MgSO_4_, 0.525 g K_2_PO_4_, 0.09 g CaCl_2_. Short-chain fatty acids (SCFA) were added to have final concentrations of acetate (33 mM), propionate (7 mM), isobutyrate, isovalerate and valerate all at 1 mM. A filter-sterilized vitamin solution was added before autoclaving to obtain final vitamin concentrations of (l^−1^ medium): 5 µg cyanocobalamin; 100 µg pyridoxine-HCl; 50 µg 4-aminobenzoic acid; 20 µg biotin (all purchased from Sigma-Aldrich Chemie GmbH). Stock solutions of different commercial folate forms (THF, M-THF, F-THF or folic acid) were prepared in NaOH solution. The stock solutions were further diluted with deionized water to a final concentration of 400 µg/l and were filtered sterilized through 0.2 µm nylon membrane filter (Infochroma AG) to be added to the medium after autoclaving. Vitamins were weighted in the dark and solutions were covered by aluminium foil and stored at 4 °C. Folate forms were tested at two doses of 50 µg/l and 200 µg/l. A negative control was used without folate addition (No B9).

Plates were filled with 1 ml of twofold concentrated bYCFA medium and 1 ml solution of each folate form or sterile water (No B9). Each diluted fecal slurry was inoculated at 0.1% (v/v; 2 µl, 10^–7^ final feces dilution on plate), and the plates were sealed with breathable seal and incubated anaerobically at 37 °C in the anaerobic chamber. Sampling was performed after 48 h incubations; 0.9 ml samples were transferred to a sterile 96-deepwell plate and centrifuged at 5,000 × *g* for 10 min at 4 °C. The supernatant was separated from the pellet and transferred into 96-deepwell plates. Pellets and supernatants were stored at -20 °C until further analysis. For each tested condition and fecal microbiota, fermentations were conducted in triplicates.

### Folate quantification using Ultra Performance Liquid chromatography (UHPLC-UV/FL)

Folate in IB extracts of folate prototrophic strains was analysed in duplicate using tri-enzyme treatment with α-amylase, hog kidney conjugase and protease as described before [24, 25] and purified by solid phase extraction using strong anion exchange columns (Bond Elut SAX, 500 mg 3 ml, Agilent Technologies, Santa Clara, USA). The cartridge was conditioned with one column volume of heptane, followed by methanol, and finally with water, and equilibrated with 10 ml of equilibration buffer (10 mM potassium phosphate buffer containing 0.1% 2,3-dimercapto-1-propanol) at pH 7.0. An aliquot of sample extract was diluted with water (1:2) and 2,3-dimercapto-1-propanol (final concentration of 0.1%), applied into SAX cartridge and washed twice with 1.5 ml of equilibration buffer. Folate was eluted slowly with 3 ml of 10% NaCl in 0.1 M acetate buffer containing 1% ascorbic acid (pH 4.5), in a 5 ml volumetric flask and filled with the elution solution.

Folate forms were determined using Waters Acquity UPLC system (Waters, Milford, MA, US) with a Kinetex PS C18 column (2.6 µm, 2.1 × 150 mm; Phenomenex, Torrance, CA, USA) at 30 °C and gradient elution with water (containing 0.7% formic acid) and acetonitrile (containing 0.7% formic acid) as mobile phases. Folate forms were detected with UV diode array detector (UV) or fluorescence detector (FL) and identified by comparing the retention times and UV spectra of the sample peaks to those of standard peaks: THF (UV and FL), M-THF (UV and FL), FFA (FL), and folic acid (UV). F-THF was detected in some samples; however, the peak was masked which made quantification unreliable. Total folate was calculated as the sum of folate forms, and concentration is expressed in ng/ml. The folate specific production is expressed as ng/10^9^ cells.

### Organic acids analysis by High Performance Liquid Chromatography with Refractive Index Detector (HPLC-RI)

Organic acids, i.e., SCFA (acetate, propionate, butyrate, and valerate), intermediate metabolites (lactate, succinate, and formate), and branched-chain fatty acids (BCFA; isobutyrate and isovalerate) were measured by HPLC-RI as reported before [26]. Briefly, feces (200 mg) were weighed and homogenized with 600 µl of HPLC eluent (100 mM H_2_SO_4_) in a tube containing one glass bead. After homogenization by vortex, samples were centrifuged for 20 min at 4 °C (6000 × g) and the supernatant was filtered through a 0.2 µm filter into a glass vial and closed with a crimp cap. For fermentation samples, samples were centrifuged for 10 min (5000 × g, 4 °C) and 0.3 ml of supernatant was filtered through a 0.20 µm nylon membrane filter plate (Macherey-Nagel AG, Oensingen, Switzerland) into a microtiter plate and further filled into the vial and closed with a crimp cap.

All samples were analyzed on Hitachi LaChrom HPLC (Merck, Dietikon, Switzerland) connected to a pre-column SecurityGuard Cartridges Carbo-H (4 × 3.0 mm; Phenomenex Helvetia GmbH, Basel, Switzerland) and a Rezex ROA-Organic Acid H + column (300 × 7.8 mm; Phenomenex Helvetia GmbH), using a refractive index detector. Samples (40 µl injection volume) were eluted with 10 mM H_2_SO_4_ at a flow rate of 0.4 ml/min under isocratic conditions at 40 °C. External standards (all Sigma-Aldrich, except valeric acid and formic acid from VWR International AG) were used for quantifying all metabolites.

### DNA isolation

DNA was extracted from 400 mg homogenized fecal sample and from 0.9 ml of fermentation sample using the FastDNA Spin Kit for Soil (MP Biomedicals, Illkirch-Graffenstaden, France), following the manufacturer’s protocol as reported before [26]. Briefly, samples were filled into 2 ml tubes containing Lysing Matrix E and homogenized (40 s, 6.0 m/s) in a FastPrep (Lucerna-Chem AG, Luzern, Switzerland) in the presence of lysis solution and sodium phosphate buffer. After the lysis, samples were centrifuged, and DNA was purified from the supernatant with a silica-based filter in a final volume of 100 µl. DNA was quantified using a Nanodrop ND-1000 spectrophotometer (Wiltec AG, Littau, Switzerland) and stored at -20 °C until further analysis.

### Quantification of total bacteria and *Roseburia intestinalis* spp. via quantitative PCR (qPCR)

Total bacteria were measured by quantifying the number of copies of the 16S rRNA gene using primers Eub_338F (5′‐ACTCCTACGGGAGGCAGCAG‐3′) and Eub_518R (5′-ATTACCGCGGCTGCTGG-3′) [27]. *R. intestinalis* qPCR targeted Roseb_intes_comEA_3, a conserved gene of *R. intestinalis* species using primers sequence 5'-GTGGACGGAGAGATGGTACG-3' and 5'-TGAATCTTGGGCTGTTTCGC-3' (Microsynth AG, Balgach, Switzerland) designed with SpeciesPrimer pipeline [28]. *R. intestinalis* primers were tested in-house against 14 other species of gut microbiota from the strain lab collection (*Anaerobutyricum hallii*, *Anaerostipes caccae*, *Anaerotigum* sp., *Bacteroides thetaiotaomicron*, *Bacteroides uniformis*, *Bifidobacterium adolescentis*, *Blautia hydrogenotrophica*, *Collinsella aerofaciens*, *Corpococus catus*, *Faecalibacterium prausnitzii*, *Lacticaseibacillus rhamnosus*, *Marvibryantia formatexigens*, *Phascolarctobacterium faecium*, *Prevotella copri*) as well as against in silico databases, and they did not show any specific amplification (data not shown). Each qPCR reaction consisted of 5 µl of 2X SensiFAST SYBR No-ROX Kit master mix (Meridian Bioscience, Cincinnati, OH, USA), 0.5 µl of each forward and reverse primer (final concentration of 0.5 µM each), 1 µl of DNA and 3 µl of nuclease free water. Reactions per sample were performed in duplicates in 96-well plates using a LightCycler 480 qPCR II system (Roche Diagnostics, Rotkreuz, Switzerland) and a two-step program consisting of 3 min of initial denaturation at 95 °C, followed by 40 cycles of 95 °C for 5 s and 65 °C for 30 s. For quantification of total bacteria, a tenfold dilution series of standards containing a linearized plasmid with the *Escherichia coli* 16S rRNA gene was included in each run. For the quantification of *R. intestinalis*, a tenfold dilution series of standards containing a conserved gene Roseb_intes_comEA_3 was included in each run. The standard amplicon was prepared using a regular PCR with the same run conditions as the qPCR. The PCR amplicon was purified using the Wizard SV Gel and PCR Clean-up System (Promega AG, Dübendorf, Switzerland) according to manufacturer’s instructions. Amplicon purity was checked with gel electrophoresis. DNA concentration was checked with Nanodrop and was converted to copy numbers per µl. PCR efficiency (%) was calculated from the slope of the standard curve of each qPCR assay. Assays with an efficiency of 65–110% (slope of 3.2–3.9) were included in the data analysis. For total bacteria quantification of fecal fermentation samples, the qPCR gene copy number was converted to cell concentration by adjusting for the median of five 16S rRNA gene copy number per bacterium based on the Ribosomal RNA Database [29].

### 16S rRNA metabarcoding and analysis

The microbial composition profile of feces and batch fermentation samples was evaluated using 16S rRNA metabarcoding. Briefly, a one-step PCR approach was used to target the V4 region using primers 515F (5′‐GTGCCAGCMGCCGCGGTAA‐3′) and 806R (5′‐GGACTACHVGGGTWTCTAAT‐3′) (Microsynth AG, Switzerland) [30]. Samples were barcoded using Nextera XT v2 indexes (Illumina, San Diego, CA, USA) and pooled in equimolar concentration. Sequencing was performed with the Illumina MiSeq platform at the Genetic Diversity Centre (ETH Zurich) using v2 chemistry supplemented with PhiX 20 pM (10%) and 250 × 2 read length. Raw data were processed using the DADA2 R package (version 1.14.1) [31], to extract exact Amplicon Sequence Variants (ASVs), as previously described [32].

For fermentation samples, phylogenetic relatedness of ASVs, and beta-diversity (weighted Unifrac distance matrices) analyses were performed using the phyloseq [33] and the DivComAnalyses packages [34] in R Studio [35].

### Statistical analysis

The statistical analysis for OD, metabolites and total bacteria was done using GraphPad Prism v.9.2.0. A two-way ANOVA was performed for data of total bacteria, ASVs, and total and individual metabolite concentrations to determine the main effects of donors and treatments. Tukey’s test (*P* < 0.05) was used for post-hoc analysis to correct for multiple comparisons. One-way ANOVA with Tukey’s test to correct for multiple comparison was performed to analyze the statistical differences (*P* < 0.05) between different treatments and to compare μ_max_ h^-1^ values between folate forms. Normality of residuals and homogeneity of variance of the data were validated using the Shapiro-Wilk test and Brown-Forsythe test, respectively. Beta diversity distance matrixes were measured by PERMANOVA analysis performed via Phyloseq (version 1.46.0) [33] in R studio [35].

## Results

### Folate production by human gut microbial prototrophs

**Fig. 1:**
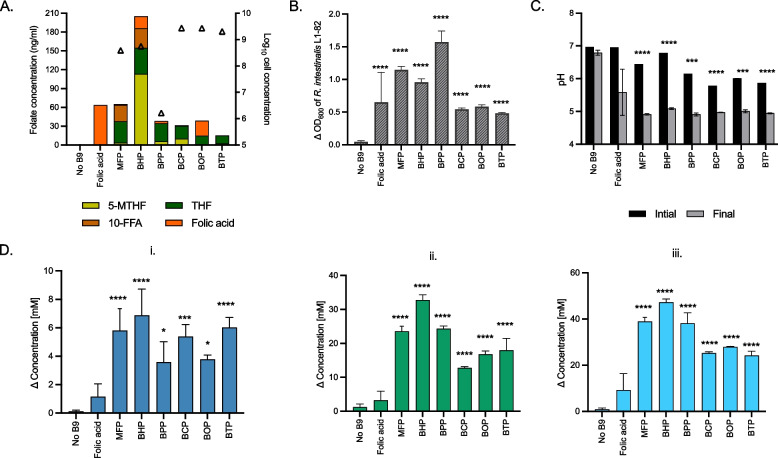
Growth and metabolism of *R. intestinalis* L1-82 grown in IB extracts from different folate prototrophs: *M. formatexigens* (MFP), *B. hydrogenotrophica* (BHP), *B. producta* (BPP), *B. caccae* (BCP), *B. ovatus* (BOP), and *B. thetaiotaomicron* (BTP). **A** Concentration of different folate forms (ng/ml, colored bars) measured in IB extracts and total bacteria counts (log10 cells/ ml, open triangles) after 60 h of growth in CDM without added folate. **B** Growth of *R. intestinalis* L1-82 in CDM with no added folate (No B9), added folic acid, or IB extracts after 48 h incubation. Delta OD corresponds to the measured OD_600_ at 48 h corrected for OD_600_ of the blank media at T0. **(C)** Initial pH and pH measured after 48 h incubation (final). **D** Production (mM) of lactate (i.), acetate (ii.), and butyrate (iii.) by *R. intestinalis* L1-82 in all treatments and control after 48 h incubation at 37 °C. Other metabolites (propionate, succinate, formate, isobutyrate, isovalerate, and valerate) were below the detection limit (1 mM). Data are averages of three biological replicates. Significant differences in Figure **B** and **D** of each treatment were compared to No B9 condition, calculated by one-way ANOVA, including Tukey’s test (* *p* < 0.05, ** *p* < 0.01, ***p < 0.001 and **** *p* < 0.0001). For Figure **C**, significant differences in each treatment between initial and final points were tested by multiple unpaired t-test, with Welch’s correction

Total folate content in the IB extracts produced by pure cultures of the six folate prototrophs followed by heat bacterial inactivation was measured by using UHPLC-FL/UV. Data showed that the selected strains produced folate within the range from 15.3 ng/ml (*B. thetaiotaomicron*; BTP) to 205.4 ng/ml (*B. hydrogenotrophica*; BHP) (Fig. 1A). Several folate forms were identified, depending on the tested strains, with THF (12.8—41.4 ng/ml) and 5-MTHF (0.2—113.3 ng/ml) being present in all samples. The concentration of 5-MTHF was low, except in BHP (113.3 ng /ml). Additionally, 10-FFA was only detected in BHP (31.5 ng/ml) and extract from *M. formatexigens* (MFP; 25.9 ng/ml). The specific total folate production ranged from 14.8 ng/10^9^ cells (BTP) to 211.3 ng/10^9^ cells (BHP) (Supplementary Figure S1). Overall, all strains identified as potential folate producers in silico were shown to produce folate in different natural forms and at various concentrations.

### Effect of IB extracts from folate gut microbial prototrophs on *R*. *intestinalis* L1-82 growth and SCFA production

To investigate whether *R. intestinalis* L1-82 utilize folate produced by prototroph gut microbes, the strain was cultivated in CDM supplemented with different IB extracts, or with folic acid. Growth across these different forms was assessed in comparison to a negative control without folate (No B9), and the production of SCFAs was evaluated. Notably, SCFA were detected in the IB extracts, with acetate being the major SCFA (ranging from 40.7 mM in BOP to 54.9 mM in BHP), followed by formate in MFP (27.9 mM) and BHP (13.3 mM), lactate in BPP (15.2 mM) and succinate in BCP (27.7 mM), BOP (24.3 mM) and BTP (24.3 mM) (Supplementary Figure S2).

The IB extracts were added at equivolume to the two times concentrated cultivation medium. After 48 h incubation, *R. intestinalis* L1-82 grew in all IB extract conditions (OD_600_ between 0.5 ± 0.01 in BTP to 1.5 ± 0.1 in BPP) compared to No B9 control for which growth was not detected (OD_600_ 0.1 ± 0.01) (Fig. 1B). In folic acid condition, the growth (OD_600_ 0.67 ± 0.5) and final pH (5.6 ± 0.7) showed high standard deviations in the biological triplicates. The pH decreased in all IB conditions (final pH from 4.9 ± 0.02 to 5.1 ± 0.02) except in No B9 (Fig. 1C). At metabolic level, *R. intestinalis* L1-82 produced significantly more acetate (ranging from + 12 ± 0.3 mM in BCP to + 32.7 ± 1.5 mM in BHP), butyrate (+ 24.2 ± 0.2 mM in BTP to + 47.2 ± 1.4 mM in BHP), and lactate (+ 3.5 ± 1.4 mM in BPP to + 6.8 ± 1.8 mM in BHP) when grown in the IB preparation of all prototrophs, compared to the No B9 condition, and to folic acid (Fig. 1D).

Overall, *R. intestinalis* L1-82 growth and activity was strongly stimulated when grown with IB extracts of the folate prototrophic gut strains.

### Effect of different folate forms on growth and metabolites of *R*. *intestinalis* L1-82

Since the tested prototrophs produced a mix of folate forms and considering the prior disparities in growth and metabolism linked to IB preparations, our objective was to examine how different pure folate forms might affect the growth and physiology of pure culture of *R. intestinalis*. Therefore, we investigated the growth kinetics of *R. intestinalis* L1-82 when cultivated for 70 h in CDM supplemented with different pure commercially available folate forms that were previously identified in IB extracts (i.e. THF, M-THF, F-THF, folic acid and FFA).

**Fig. 2:**
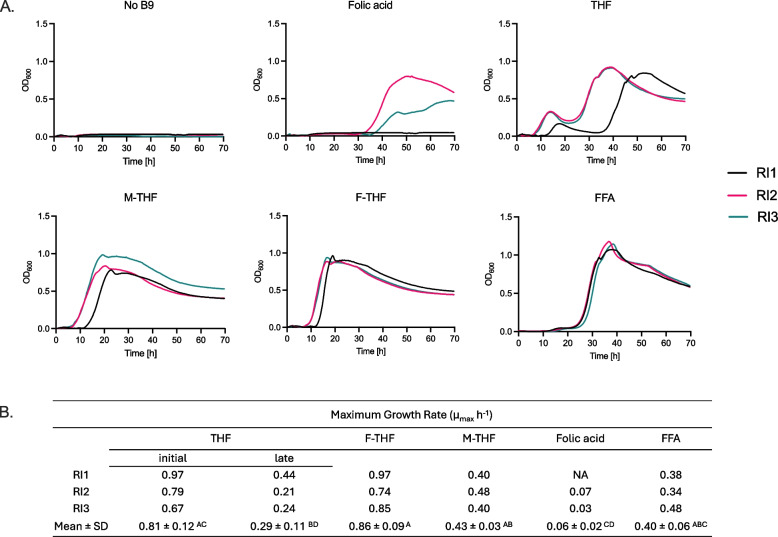
Growth kinetics of *R. intestinalis* L1-82 with different folate forms. **A** Growth profiles for three biological replicate RI1, RI2 and RI3 (each with two technical replicate). The OD at 600 nm was measured every 20 min during 70 h of incubation. F-THF = formyl-tetrahydrofolate, M-THF = methyl-tetrahydrofolate, THF = tetrahydrofolate, FFA = 10-formylfolic acid, No B9 = without added folate. **B **Maximum growth rates (µ_max_, h.^−1^) calculated from the exponential growth period of biological replicates. Mean represents an average of three biological replicates and standard deviation. NA, not analyzed. Different letters indicate significant differences between the μ_max_ of different folate forms calculated by one-way ANOVA and Tukey’s multiple comparison test (*p* < 0.05)

*R. intestinalis* L1-82 grew with all the tested folate forms, but not in the absence of folate (Fig. 2A). Maximum growth was observed with F-THF supplementation, with maximum OD_600_ measured after 17 h (OD_max_ = 0.9 ± 0.1), and the highest maximum specific growth rate (µ_max_ = 0.86 ± 0.09 h^−1^) among treatments. In contrast, M-THF showed an intermediate specific growth rate (µ_max_ = 0.43 ± 0.03 h^−1^) with OD_max_ of 0.9 ± 0.1 measured after 21 h, and a similar intermediate growth rate was also measured for FFA (µ_max_ = 0.40 ± 0.06 h^−1^). Interestingly, a biphasic growth was observed with THF, with the initial growth phase starting after approx. 8 h (µ_max_ = 0.81 ± 0.12 h^−1^) followed by a decrease of OD possibly suggesting lysis period. A second late growth phase in THF started after approx. 25 h (µ_max_ = 0.29 ± 0.11 h^−1^). Growth in folic acid was not reproducible, and started only after 30 h for two repeats (µ_max_ = 0.06 ± 0.02 h^−1^) whereas no growth was detected for the third one. The growth with folate supplementation resulted in the production of mainly acetate and butyrate, and an expected low lactate concentration, and was similar in all folate treatments after 70 h incubation (Supplementary Figure S3).

**Fig. 3:**
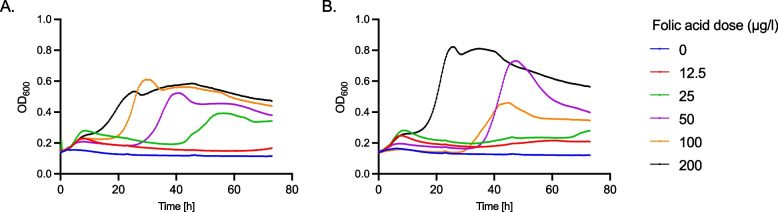
Growth curves of *R. intestinalis* L1-82 with different doses of folic acid (µg/l). For each dose, the average of three technical replicates is plotted from 0 to 70 h growth for biological replicate (**A**) RI1, and (**B**) RI2. The OD at 600 nm was measured automatically every 20 min during 70 h

To evaluate whether folic acid was limiting in the tested medium, the effect of different doses of folic acid (0 to 200 µg/l) was then tested on the growth of *R. intestinalis* L1-82. We observed that growth was strongly dependent on the dose, with the highest µ_max_ and OD_max_ and the shortest lag time measured for the highest tested level of 200 µg/l of folic acid (Fig. 3 and Supplementary Figure S4).

Overall, our results confirmed that the natural folate forms, particularly M-THF and F-THF, strongly promoted the growth of *R. intestinalis* L1-82. On the contrary, folic acid exhibited a dose dependent effect and a high variability. This underscores the potential benefits of utilizing natural folate forms for fostering *R. intestinalis* growth.

### Fecal donor microbiota and metabolic profiles of five healthy donors

**Fig. 4:**
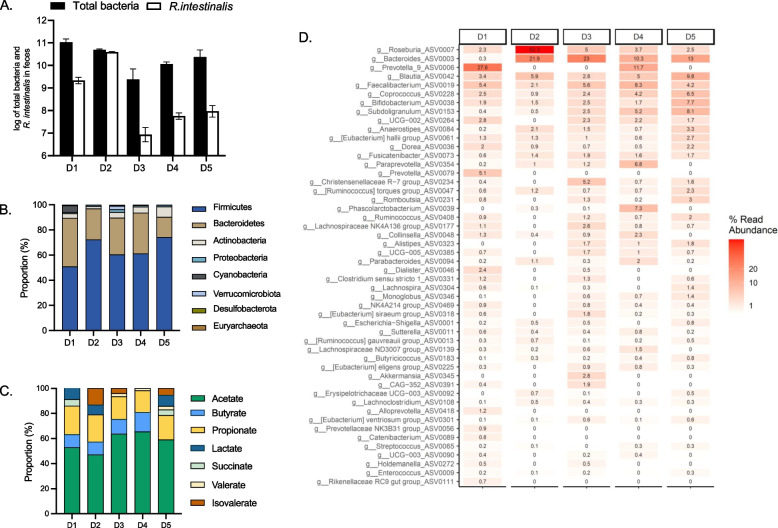
Fecal microbiota composition and metabolite profile of five healthy adult fecal donors (D1 to D5). **A** Total bacteria (log total bacteria/g feces) and *R. intestinalis* (log cell/g feces) quantified using qPCR. **B** Relative abundance of fecal microbial communities of each donor at phylum and (**C**) at genus level measured with 16S amplicon sequencing. **D** Metabolite proportion in feces of each donor. For all donors, formate was below detection limit (1 mM)

We next sought to preliminary explore the effects of different folate forms on the growth and composition of gut microbial communities and metabolite production during 48 h batch fermentations inoculated with fecal suspension from five healthy adult donors. The average concentration of total bacteria measured by qPCR in fecal donor samples ranged from 9.3 to 11 log cell number per gram of feces across donors. *R. intestinalis* was present in all fecal microbiota at concentration ranging from log 6.9 in D3 to 10.5 in D2 cells/g feces (Fig. 4A). The *Firmicutes* phylum was the most abundant in the donor samples, representing 52% in D1 to 75% in D5, while *Bacteroidetes* was most abundant in D2 (49%) and lowest in D5 (14%) (Fig. 4B). At genus level, *Bacteroides* was the most abundant genus in D3 (23%), D4 (10%) and D5 (13%), while *Prevotella* was dominant in D1 (28%), and *Roseburia* in D2 (52%). In line with qPCR, all donors exhibited varying levels of *Roseburia* from 2% in D1 to 52% in D2 (Fig. 4D). From the analysis of SCFA, acetate was detected as the major metabolite (47% in D2 to 65% in D4), followed by propionate (17% in D4 to 23% in D1) and butyrate (10% in D1 and D2, to 15% in D4), while no butyrate was detected in D5 (Fig. 4C). Even if in low number, the donors tested provided a good representation and spread of the target population of adult gut microbiota allowing to further evaluate different folate forms and doses on complex gut microbial communities.

### Impact of different folate forms on fecal community growth and metabolite production

**Fig. 5:**
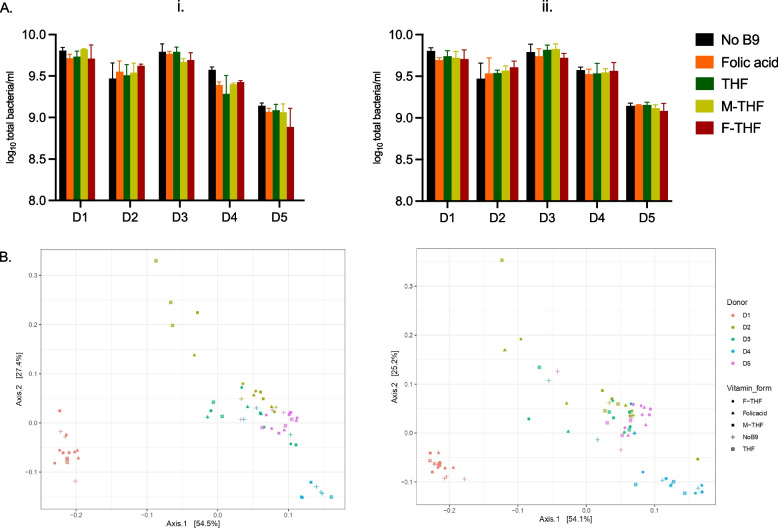
Total bacteria concentrations (**A**) and principal-coordinate analysis plots (PCoA) of weighted Unifrac distance matrices (**B**) after 48 h fecal batch fermentation of five microbiota (D1 to D5) with different folate forms (THF, folic acid, F-THF, M-THF) at two doses of 50 µg/l (i.) and 200 µg/l (ii.) and no folate addition (No B9). Total bacteria data are means and standard deviations of three biological replicates

Using fecal batch fermentations of the five donors, we investigated the effect of a low (50 µg/l) and high (200 µg/l) dose of different folate forms on growth and metabolism after 48 h incubation. Surprisingly, neither of the folate forms nor doses impacted total bacteria concentration after 48 h of batch incubation (Fig. 5A). Furthermore, we did not detect significant changes in the microbiota community beta diversity for the different folate forms compared to the no folate control using PERMANOVA analysis (Fig. 5B). Lastly, no significant effect of folate forms and doses was observed for the measured metabolites for D1, D2 and D4 while differences were observed for D3 and D5 under low folate dose (Supplementary Figure S5).

**Fig. 6:**
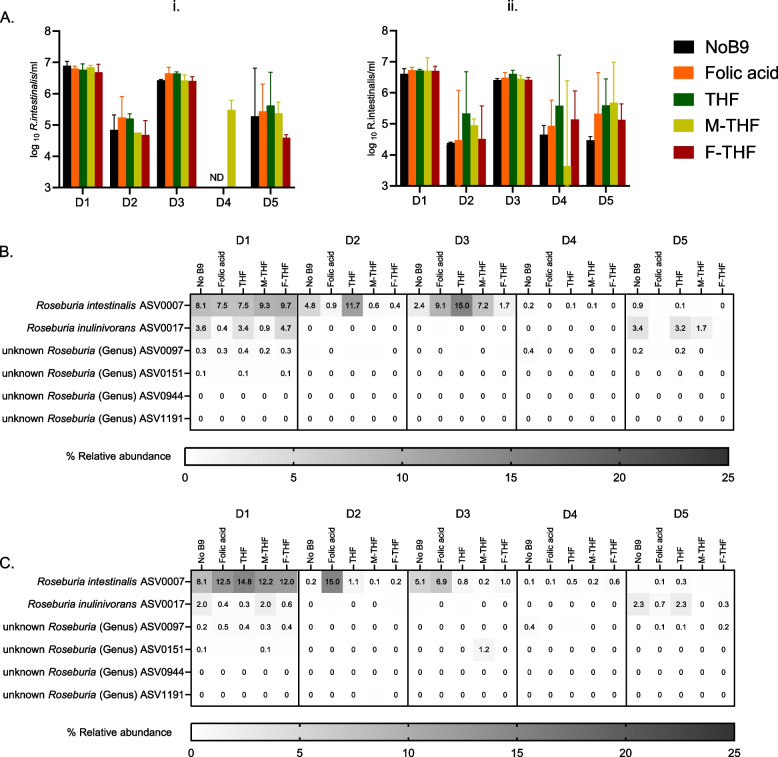
Concentration and relative abundance of *Roseburia intestinalis* in all tested conditions. **A ***Roseburia intestinalis* quantified by qPCR after 48 h fecal batch fermentations with 50 µg/l (i.) and 200 µg/l (ii.) of five donors (D1-D5). Values are mean of independent triplicates. Missing values were not detected. Heatmaps represent relative abundance (%) of *Roseburia* ASVs after rarefication in the samples with 50 µg/l (**B**) and 200 µg/l (**C**) folate dose. Values are presented as a means of triplicates

In line with that was observed for total bacteria concentration, overall neither folate forms nor their doses impacted significantly total cell numbers of *R. intestinalis* and the small variations observed were due to the donor’s effect and not the applied treatments (Fig. 6A). In addition, within each donor, total cell numbers of *R. intestinalis* were not significantly different among tested different folate forms as well as between the two doses, except in D4 since *R. intestinalis* was undetected in this donor.

Furthermore, we investigated the effect of different forms of folate on the abundance of ASVs belonging to the *Roseburia* genus, especially *R. intestinalis* ASV0007, as it was the only ASV assigned to *R. intestinalis* species (Fig. 6B and C). Our analysis showed that overall neither high or low dose of different folate forms led to significant changes in the abundance of *R. intestinalis* ASV0007 and the same applied when testing different folate forms for each donor (Supplementary Table 1).

## Discussion

The main goal of our study was to determine if and how different forms of folate – synthetic folic acid and forms produced by gut microbes – can support the growth of the folate auxotrophic and butyrate-producing *R. intestinalis* and modulate the human gut microbiota composition and activity profiles.

First, we confirmed and quantified the production of different folate forms of all tested predicted folate prototrophic gut strains. These findings confirmed the earlier in silico predictions of folate production by these strains [7, 11]. Notably, we identified for the first time *B. hydrogenotrophic* (205.4 ng/ml of total folate) and *M. formatexigens* (65.2 ng/ml of total folate) as high folate producers among the tested strains. Further, our data demonstrated that all tested prototrophic strains primarily produced THF and M-THF, which are the most common microbially-produced folate forms previously detected in vitro [10, 36]. Recently, it has been demonstrated in fecal incubations that human gut microbial communities could produce folate [37]. However, to date, only a few gut microbes have been demonstrated to produce folate but none of the studies identified the produced folate forms. These include species from the *Bifidobacterium* genus and *Akkermansia muciniphila* [10, 38, 39].

In this study, we demonstrated that folate produced by selected gut microbes supports the growth of butyrate producing *R. intestinalis* L1-82. Our findings align with a recent study that highlighted the consumption of folate produced by other gut bacteria i.e., *Coprococcus* sp. ART55/1, *Streptococcus thermophilus* CNCM I-3862 and *Bifidobacterium bifidum* CNCM I-3650, by the folate auxotrophic *Roseburia* species [16]. However, the authors utilized a synthetic community of five folate auxotrophs in combination with one prototroph to explore the cross feeding of folates, resulting to competition-induced loss of several auxotrophic strains. The folate quantity and forms for the prototrophic strains tested were also not investigated. Here, using IB extracts of individual folate prototrophs we could confirm and quantify the different folate forms and test their effects on the auxotrophic strain of *R. intestinalis*, independently of microbial competition.

To investigate further the impact of the different folate forms detected in the IB extracts, we monitored the growth of *R. intestinalis* L1-82 on commercially available natural forms of folate provided individually and monitored the growth curve and metabolite production. Our data showed that natural folate forms resulted in higher and faster growth as compared to the synthetic form of folic acid. Interestingly, our findings align with clinical trial results, which indicated that the human gut microbiota have greater capacity to convert dietary folate (tested as 5-FTHF) to 5-MTHF, when compared to folic acid [40], a process where folic acid is reduced to dihydrofolate and then further to THF [41]. This might suggest that the biphasic growth we observed with folic acid is associated with the delayed conversion of folic acid to THF, further highlighting the critical role that different folate forms have on microbial growth.

Lastly, we performed in vitro fecal batch fermentations to investigate the effect of different folate forms on the growth and metabolism of the gut microbiota, with a particular focus on the *R. intestinalis*. Unexpectedly, we did not observe any significant impact of different folate forms and doses on the community composition and main metabolites production. This contrasts with a previous study that reported an increase in the number of observed species with different doses of folic acid [42]. On the other hand, in agreement with our data, a study in rats reported that folic acid supplementation did not impact the abundance of the two most abundant phyla, *Firmicutes* and *Bacteroidetes* [43]. However, a threefold increase of the *Actinobacteria* phylum abundance was reported by the authors, in contrast to our study. Additionally, our study found that a high dose of folate did not result in an increased abundance of *Roseburia* genus, compared to the low dose tested. Recently, we have also reported that gut microbial communities produce B12 during in vitro fecal batch fermentation in absence of B12 in the medium while different forms of B12 had no impact on total bacterial growth, community richness, diversity, and total metabolite production [26]. Overall our in vitro data suggested that healthy human adult gut microbial communities have the capacity to produce both B12 and B9 at levels fulfilling most of their own requirements. However, our folate data should be expanded by testing additional fecal microbiota representative of a broader population.

To sum up, our study demonstrated and quantified the actual production of different folate forms by the selected gut prototrophs and further showed that *R. intestinalis* L1-82 can efficiently use microbially- produced folates for growth and metabolism. These findings may be important to adjust the growth medium to produce *R. intestinalis* as second-generation probiotic. However, it will be interesting to further investigate if such an outcome is strain dependent. Moreover, future studies should also focus on evaluating the different forms of folate produced by gut microbes for their bioavailability and absorption by the host. Although there is sufficient evidence that microbially produced folate is absorbed in the large intestine [36, 44], certain forms are not bioactive for humans and must be converted into active forms by gut bacteria before it can be used by the human host [45].

## Conclusions

In conclusion, this study reveals that *R. intestinalis* L1-82, a beneficial butyrate-producing member of the gut microbiota, uses different forms of folate produced by folate prototrophs of the human gut. Interestingly, the different folate forms impacted differently its growth kinetics. In the complex community gut microbiota, the effects of folate forms and doses on composition and metabolism varied across the donors tested. Our observations suggest that gut microbiota produce folate primarily to fulfil its own requirement and that the dietary folate forms at the tested levels may only have limited effects, if any, on the human gut microbiota in vivo*.* Further research with a larger range of fecal donors is necessary to fully determine the possible long-term impact of folate sustained supplementation on the gut microbiota and gut health.

## Supplementary Information

Supplementary Material 1.

Supplementary Material 2.

Supplementary Material 3.

Supplementary Material 4.

Supplementary Material 5.

Supplementary Material 6.

## Data Availability

Sequence data that support the findings of this study have been deposited in the repository NCBI, using the identifier number BioProject ID: PRJNA1114027: https://www.ncbi.nlm.nih.gov/bioproject/PRJNA1114027.
