# Supplementary figures and images for: Microbially-produced folate forms support the growth of Roseburia intestinalis but not its competitive fitness in fecal batch fermentations
Source: BMC Microbiol. 2024 Sep 28;24:366. doi: 10.1186/s12866-024-03528-6 (PMC11438134; doi:10.1186/s12866-024-03528-6)

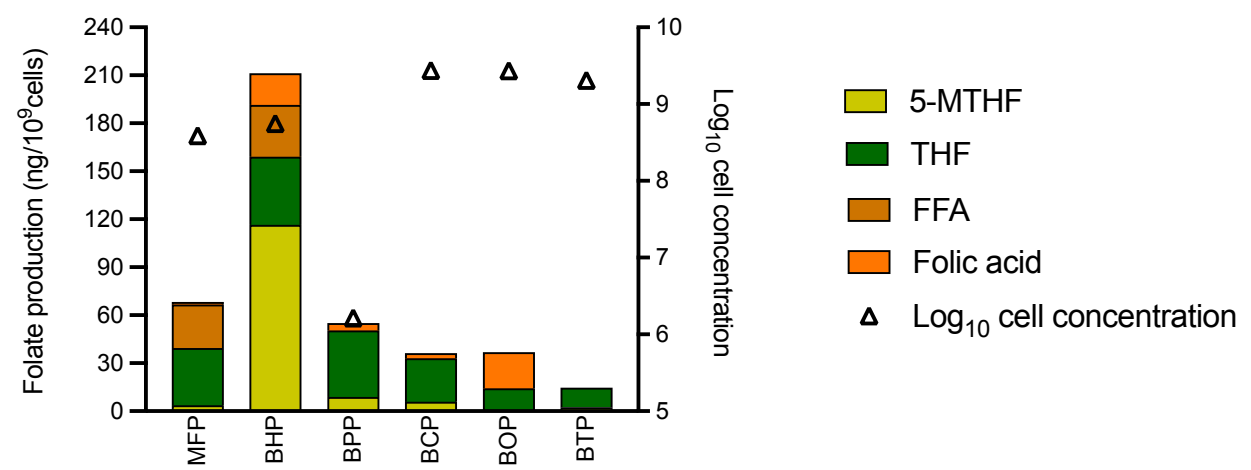

Supplement: Supplementary file 1 — Supplementary Material 1. [file 12866_2024_3528_MOESM1_ESM.pdf]

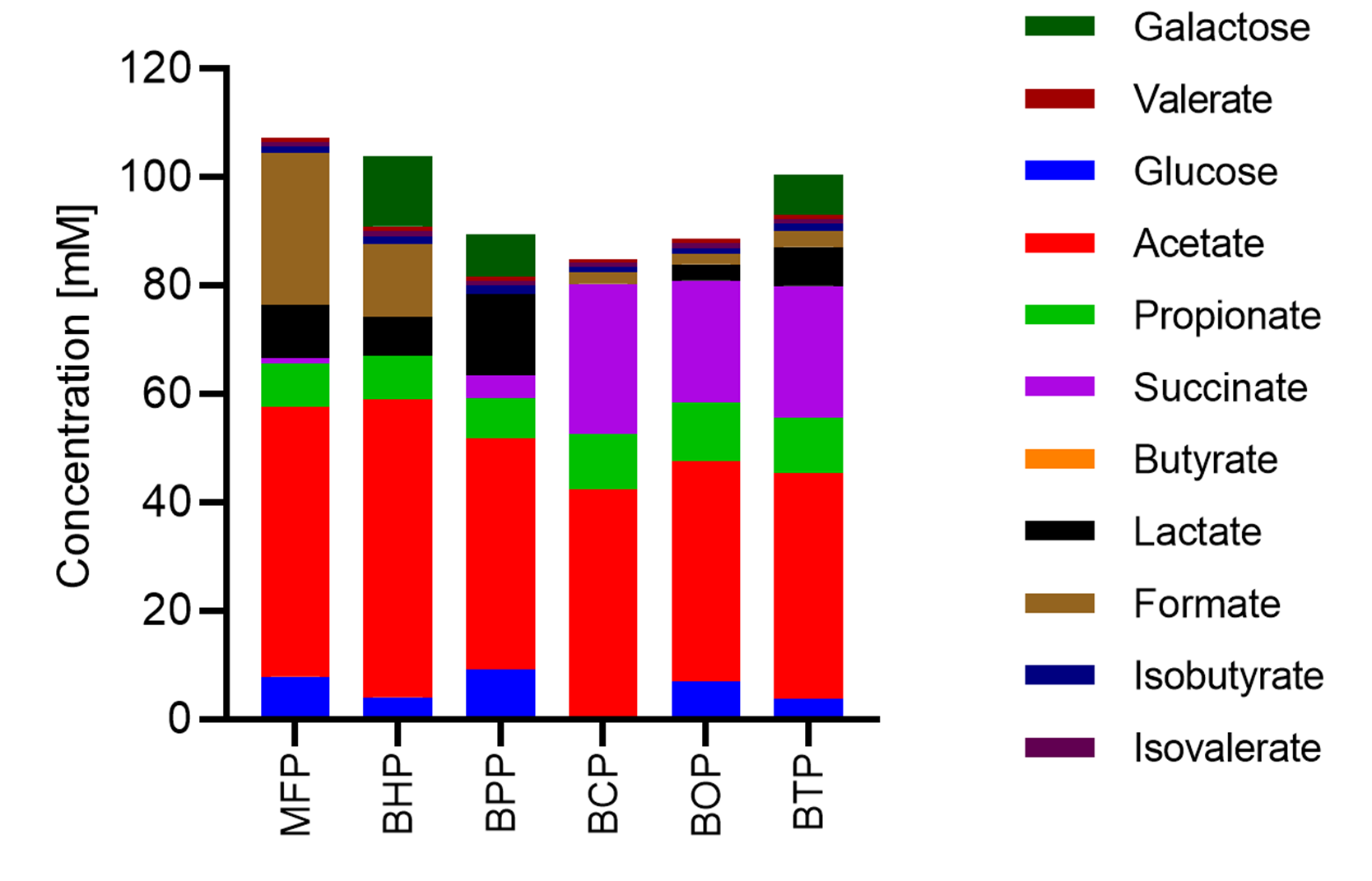

Supplement: Supplementary file 2 — Supplementary Material 2. [file 12866_2024_3528_MOESM2_ESM.tif]

Acetate

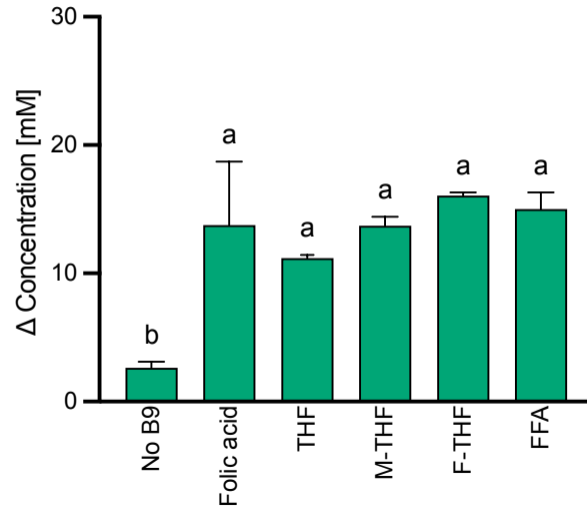

Butyrate

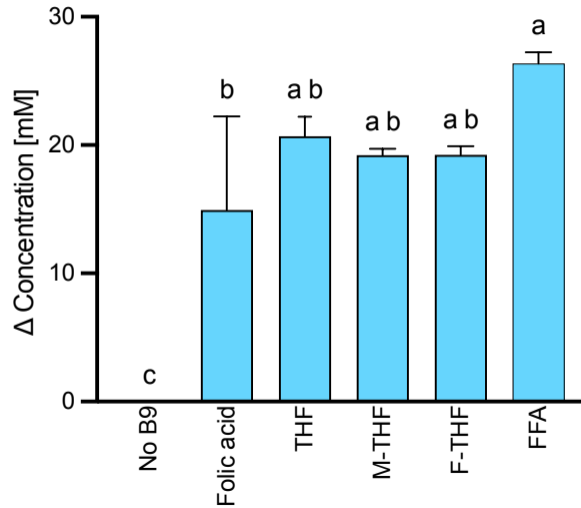

Lactate

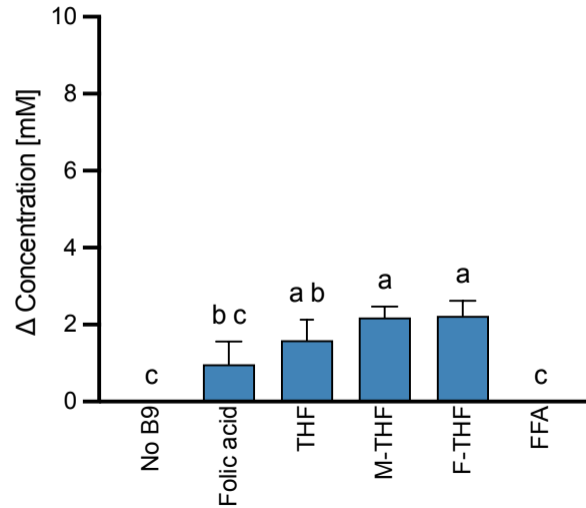

Supplement: Supplementary file 3 — Supplementary Material 3. [file 12866_2024_3528_MOESM3_ESM.pdf]

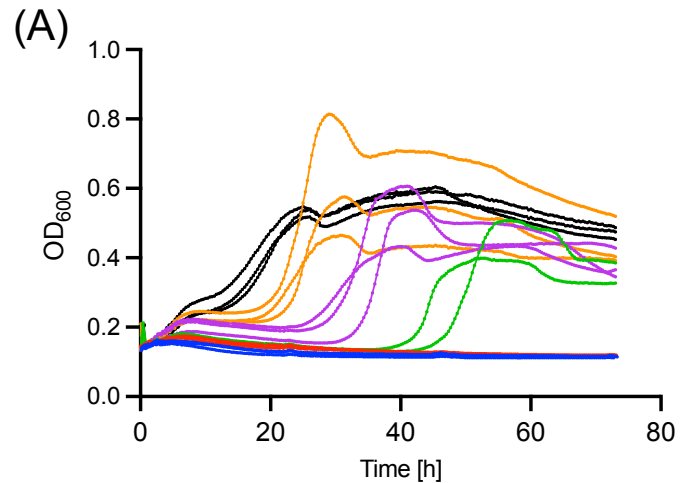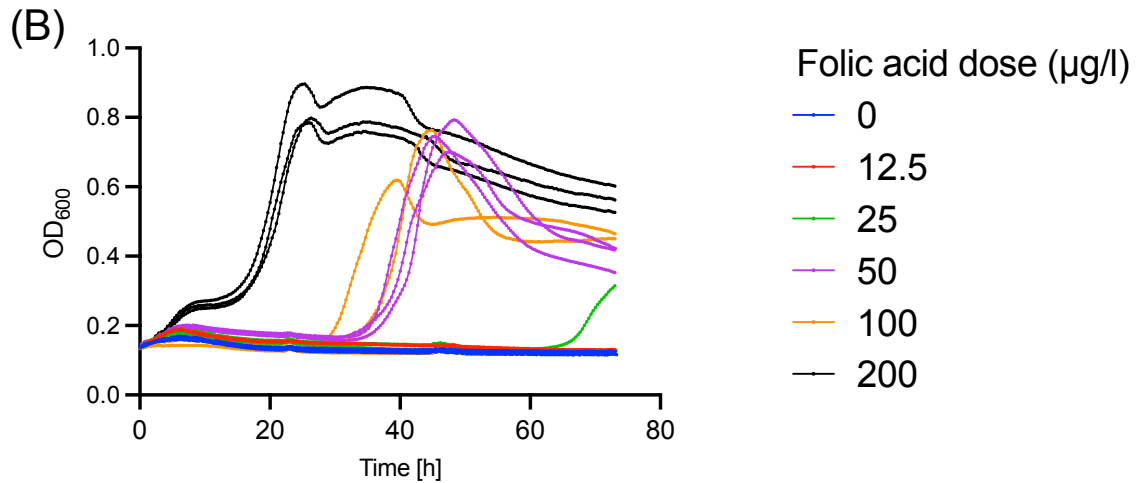

Supplement: Supplementary file 4 — Supplementary Material 4. [file 12866_2024_3528_MOESM4_ESM.pdf]

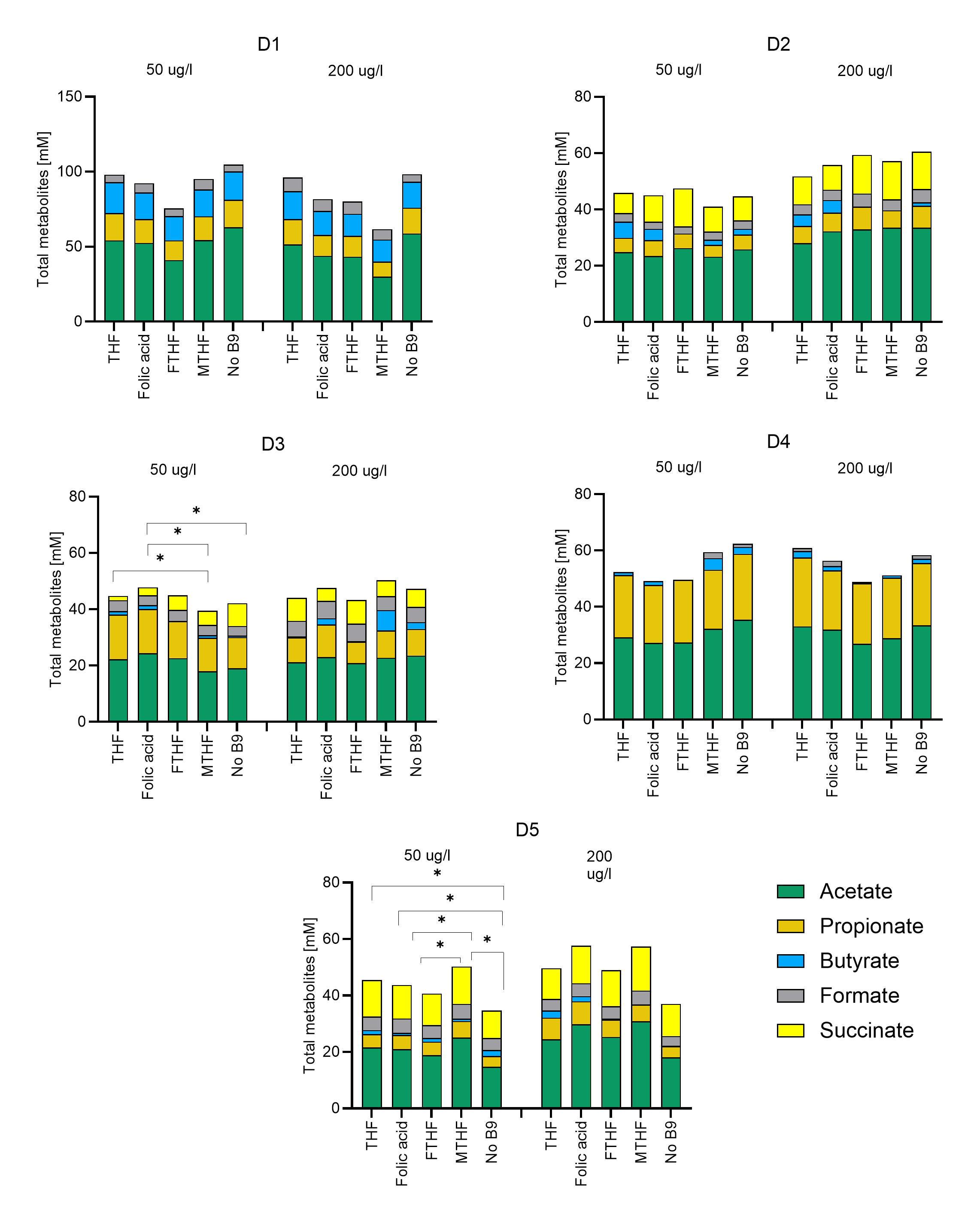

Supplement: Supplementary file 5 — Supplementary Material 5. [file 12866_2024_3528_MOESM5_ESM.tif]
